# Incidence of hospital contacts with acute kidney injury after initiation of second-generation antipsychotics in older adults: a Danish population-based cohort study

**DOI:** 10.1007/s00228-022-03339-6

**Published:** 2022-05-31

**Authors:** Reeha Sharon, Theis Lange, Mia Aakjær, Sarah Brøgger Kristiansen, Morten Baltzer Houlind, Morten Andersen

**Affiliations:** 1 Pharmacovigilance Research Center, Department of Drug Design and Pharmacology, Faculty of Health and Medical Sciences, University of Copenhagen, Copenhagen, Denmark; 2 Section of Biostatistics, Department of Public Health, University of Copenhagen, Copenhagen, Denmark; 3 Department of Clinical Research, Copenhagen University Hospital, Hvidovre, Denmark; 4 The Capital Region Pharmacy, Herlev, Denmark; 5 Department of Drug Design and Pharmacology, University of Copenhagen, Copenhagen, Denmark

**Keywords:** Second-generation antipsychotics, SGA, Olanzapine, Quetiapine, Risperidone, Acute kidney injury, AKI

## Abstract

**Purpose:**

To investigate the association between acute kidney injury (AKI) and use of second-generation antipsychotics (SGA) in older adults.

**Methods:**

In a population-based cohort study using Danish national registries, new users of SGAs (aged ≥ 65) were identified during 2005–2015. Each SGA user was matched to 10 population controls on age, sex, and the SGA initiation date. The outcome was incident AKI within 90 days after the index date. Cox regression was used to estimate hazard ratios (HRs) with 95% confidence intervals (CIs), adjusting for potential confounders.

**Results:**

In the study, 36,581 new SGA users and 365,810 controls were included. The 90-day incidence rate of AKI was 4.38 and 1.70 per 1000 person-years among SGA users and controls, respectively, corresponding to a crude HR of 2.57 (1.79–3.68). The fully adjusted HR (aHR) was 1.43 (0.89–2.27) for all SGAs. The risk differed among individual drugs with aHRs for olanzapine 3.50 (1.20–10.23), quetiapine 1.62 (0.81–3.26), and risperidone 0.68 (0.28–1.64). In sensitivity analyses, the aHR declined to 1.24 (0.95–1.61) at 1-year follow-up.

**Conclusions:**

Olanzapine use was associated with a significantly increased 90-day AKI risk. For quetiapine, the risk was elevated but not significant, and risperidone had no association. CIs were wide and confounder adjustment largely impacted the estimates. Main limitations included residual confounding and incomplete recording of AKI diagnoses.

**Supplementary information:**

The online version contains supplementary material available at 10.1007/s00228-022-03339-6.

## Introduction

Second-generation antipsychotics (SGA) are used for schizophrenia, treatment-resistant depression, organic delirium or hallucinations, and maintenance therapy of behavioral and psychological symptoms in dementia in older adults, e.g., agitation, aggression, and psychotic symptoms [1, 2].

The use of antipsychotics in older people is discouraged because of a high risk of adverse effects [3], limited evidence of efficacy [4], and extrapolated evidence from the younger population [5]. The most severe adverse effects include cerebrovascular or cardiovascular events or even death [6–8]. Drug safety warnings have been issued against the use of SGAs in older adults, particularly in dementia [2], and such warnings may have influenced prescribing practice. In Denmark, the prevalence of antipsychotic use in older adults with dementia decreased from 31.3% in 2000 to 20.4% in 2012 [9], which is, however, still considerably high use. This continued use of antipsychotics could be due to a lack of other treatment options [3].

Older adults are at a higher risk for developing acute kidney injury (AKI) compared to younger adults, due to age-related kidney function decline, chronic kidney disease (CKD) and other comorbidities, increased levels of chronic inflammation, and use of more medication [10]. The pathophysiology of AKI is multifactorial and complex [11]. Outpatient AKI is caused by infections, dehydration, and some medications, e.g., non-steroidal anti-inflammatory drugs (NSAIDs), lithium, anti-hypertensives, and cardiovascular or anti-cancer drugs [12]. Inpatient AKI can be due to the same factors, as well as to hospital-specific nephrotoxic exposures, e.g., vancomycin and aminoglycosides [13], and is most frequently encountered in patients with several comorbidities [14]. In more severe cases of AKI, dialysis is required to replace kidney function. The crude incidence of dialysis requiring AKI in Denmark increased from 143 per million in 2000 to 366 per million in 2006 and remained the same until 2012 [15]. This also reflected increased dialyses in patients aged > 75 [15]. AKI is common in hospital admissions among older adults with a prevalence of 9.7–12.1% [16, 17]. Among intensive care patients, 1-year mortality rates among older adults with AKI are much higher (48.7–57.4%) than in patients without AKI (22.1%) [18]. Thus, drug-induced AKI could potentially contribute to both morbidity and mortality in SGA users.

Although older adults are considered at a higher risk for AKI in general, only a few studies have investigated the association between SGA use and AKI in older adults and the evidence is conflicting [19–21]. A recent study in Denmark found an association between SGAs and CKD [22], but did not investigate the sudden onset of AKI. We hypothezised that initiation of SGA treatment in older adults could lead to an increased incidence of AKI. We performed a cohort study using data from national healthcare registers, with the primary aim of investigating the short-term incidence of AKI in older SGA initiators compared to the incidence in the general population. A secondary aim was to investigate possible differences in risk among the SGAs.

## Method

### Data sources

We performed a nationwide cohort study by linking several Danish healthcare and administrative registers using the unique Danish civil registration number assigned to all residents [23]. Information on drug dispensings from community pharmacies was retrieved from the Danish National Prescription Registry (37) using Anatomical Therapeutic Chemical classification (ATC) codes [24] (Suppl. Table 1). Information regarding hospitalizations, diagnoses, and procedures were retrieved from the Danish National Patient Registry [25] and deaths from the Cause of Death Register [26]. Diagnoses were coded according to the 10th edition of the International Classification of Diseases (ICD) and procedures according to the Nordic Classification of Surgical Procedures (NCSP) [27] and the Danish Health Care Classification System (Sundhedsvæsenets Klassifikations System (SKS)) [28] (Suppl. Table 2). Migration and residence status were retrieved from the Danish Civil Registry [23]. All register-based data were processed using the Nordic Common Data Model (NCDM) [29].

This study was part of a project that aims to evaluate drug safety and effectiveness of medications in patients aged 65 years or older [30]. The project was approved by the Danish Data Protection Agency through the University of Copenhagen (ref. no. 514–0249/18–3000), the Danish Health Data Authority (FSEID-00003916), and Statistics Denmark (project no. 707278).

### Study population and follow-up

The base study population included all persons aged ≥ 65 who were alive and resident in Denmark between 2005 and 2015 (1,553,876 persons). Within this population, we identified all new users of SGAs, without a preceding antipsychotic (first-generation antipsychotic or SGA) within the last 2 years before the first dispensing in the study inclusion period. We included all SGAs marketed in Denmark during the study period (Suppl. Table 1), of which the three most-used drugs were olanzapine, quetiapine, and risperidone. The date of the first dispensing was the index date. For each SGA user, we selected 10 controls from the base study population by matching on index date, age (same birth year), and sex. All individuals were allowed to dynamically move from the SGA user cohort to the control cohort and vice versa if they fulfilled cohort entry criteria of inclusion and exclusion. The time windows for assessment of inclusion and exclusion criteria, covariates, and follow-up are depicted using a graphical study design (Suppl. Fig. 1) [31].

**Fig. 1:**
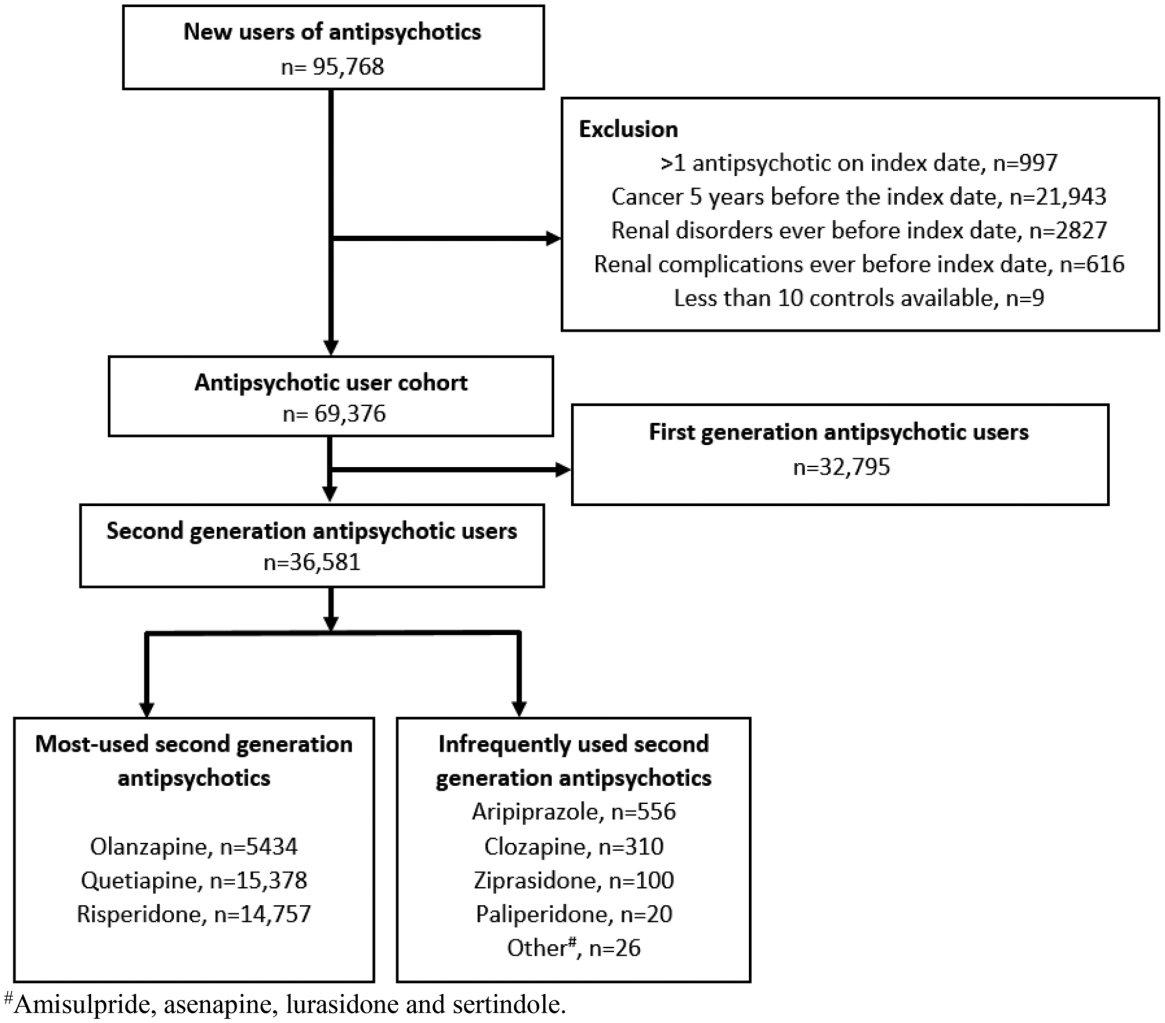
Study flow chart for selection of the second-generation antipsychotic user cohort

Patients with a history of any renal disease or related interventions and procedures [32] or renal complications secondary to another disease (e.g., diabetes and hypertension) during the past 10 years were excluded. AKI is also a known complication to malignant diseases [15, 33]; hence, people with a cancer diagnosis 5 years prior to cohort entry were excluded (Suppl. Table 2). Additionally, to enable individual drug comparisons, patients with more than one SGA dispensed on the index date were also excluded. The same exclusion criteria were applied to the controls during the sampling process. After performing the exclusions, the cohort consisted of 36,581 SGA users. A flowchart is shown in Fig. 1. All individuals in the cohort were followed until 90 days for the occurrence of AKI or censored in case of death or migration. The 90-day follow-up approach (similar to other studies) was selected to focus only on potential acute adverse events [19]. We allowed for follow-up until the end of the study period in 2016.

### Outcomes

The outcome was 10-year incident hospital contacts with a diagnosis of AKI (ICD-10 codes: N00 [acute nephritic syndrome] and N17 [acute kidney failure]) within 90 days of initiation of a SGA. We included both outpatient contacts and inpatient admissions.

### Comorbidity and co-medication

In the statistical analyses, we adjusted for potential confounders contained in the Danish National Patient Registry and the Danish National Prescription Registry. Co-medications included were NSAIDs, lithium, anti-hypertensives, other cardiovascular drugs, anti-diabetics, alcohol dependency drugs, anti-epileptics, anxiolytics, and antidepressant drugs dispensed before the index date (Suppl. Table 1). Comorbidities were alcohol abuse, cardiovascular diseases, cerebrovascular diseases, diabetes, hepatic diseases, hypertension, obesity, Parkinson’s disease, and vascular diseases from both inpatient and outpatient hospital encounters (Suppl. Table 2). The covariate recent hospitalization was defined as hospital discharge in the last 30 days before the index date. The time windows for each of the covariates are described in Suppl. Fig. 1.

### Statistics

We calculated the incidence rate (IR) of AKI. A Cox proportional hazards regression model was used to estimate crude and adjusted hazard ratios (aHR) with 95% confidence intervals (CIs). In a partially adjusted model, we included covariates for comorbidities and co-medications. In the fully adjusted model, the additional covariate was recent hospitalization. A sandwich covariance estimator accounted for intra-cluster dependence, as the same individual could be selected as control multiple times [34]. All data management and analyses were performed with the NCDM analytics framework (Suppl. Fig. 2), using modular SAS programs (version 9.4; SAS Institute, Inc., Cary, NC, USA) and Stata (StataCorp. 2019. Stata Statistical Software: Release 16. College Station, TX: StataCorp LLC.).

### Sensitivity analyses

We performed pre-planned sensitivity analyses with follow-ups of 180 days and 1 year to check for changes in risk over time [19]. Due to inadequate sensitivity to detect AKI diagnosis using ICD-10 codes [17, 35], additional analysis was performed with dialysis as outcome using SKS codes: BJFD00 (acute intermittent hemodialysis), BJFD01 (acute peritoneal dialysis), BJFD02 (continuous renal replacement therapy), and BJFD0 (unspecified acute dialysis) [15].

## Results

The matched cohort comprised of 36,581 SGA users and 365,810 controls. The most used SGAs were quetiapine (42.0%) and risperidone (40.4%), followed by olanzapine (14.9%). The cohort had a higher proportion of females (61.3%) than males and individuals ≥ 80 years constituted the most frequent age group. Baseline characteristics of the study population are presented in Table 1. Neuropsychiatric comorbidities were found to be higher in SGA users as compared to controls, most notably dementia (35.0% vs 4.5%), depression (16.4% vs 2.4%), and Parkinson’s disease (10.3% vs 3.0%). Also, the psychotropic co-medications were more prevalent among the SGA users, including antidepressants (56.3% vs 13.8%) and anxiolytics (27.0% vs 7.4%). A substantially larger proportion of SGA users than controls were hospitalized in the last month prior to cohort entry (27.4% vs 2.8%). The SGA users also had more somatic comorbidities than controls, such as stroke (18.2% vs 8.3%) and alcohol abuse-related disorders (4.4% vs 0.8%) (Table 1).

**Table 1:** Baseline characteristics of 36,581 SGA users and 365,810 controls during 2005–2015, including demographics, comorbidities, psychiatric conditions, and co-medications

	SGA users* N	**%**	**Controls** ***N***	**%**
***N***	36,581	100	365,810	100
**Sex;** female	22,419	61.3	224,190	61.3
**Age group** (years)				
65–69	4569	12.5	45,690	12.5
70–74	5209	14.2	52,090	14.2
75–79	6078	16.6	60,780	16.6
80–84	7412	20.3	74,120	20.3
85–89	7093	19.4	70,930	19.4
90 +	6220	17.0	62,200	17.0
**Comorbidities**				
Diabetes	4287	11.7	36,134	9.9
Hypertension	27,472	75.1	257,060	70.3
Cardiovascular diseases	23,768	65.0	208,343	57.0
Acute myocardial infarction	1294	3.5	10,098	2.8
Arrhythmia	4838	13.2	33,415	9.1
Congestive heart failure	2354	6.4	16,864	4.6
Cerebrovascular diseases/stroke	6670	18.2	30,452	8.3
Vascular diseases	1580	4.3	12,914	3.5
Atherosclerosis	975	2.7	6726	1.8
Alcohol abuse	1590	4.4	3002	0.8
Obesity	3134	8.6	20,446	5.6
Liver disease	445	1.2	2025	0.6
**Psychiatric conditions and others**				
Alzheimer’s disease	1488	4.1	1974	0.5
Anxiety	913	2.5	1031	0.3
Bipolar disorder	1029	2.8	362	0.1
Dementia	12,787	35.0	16,436	4.5
Depression	6002	16.4	8807	2.4
Mood disorders	348	1.0	448	0.1
Schizophrenia and schizoaffective disorders	1795	4.9	355	0.1
Epilepsy	848	2.3	2696	0.7
Parkinson’s disease	3776	10.3	10,849	3.0
**Recent hospitalizations**				
Any hospitalizations	10,034	27.4	10,390	2.8
Psychiatric	3406	9.3	59	0.02
Surgery	1692	4.6	3781	1.0
Medical	6082	16.6	6793	1.9
**Co-medications**				
Antiepileptics	3298	9.0	10,543	2.9
Antidepressants	20,607	56.3	50,547	13.8
Anxiolytics	9882	27.0	27,030	7.4
NSAIDs	7186	19.6	66,162	18.1
Lithium	1146	3.1	1185	0.3

*N/A* not applicable, *NSAIDs* non-steroidal anti-inflammatory drugs, *SGA* second-generation antipsychotics

^*^Amisulpride, aripiprazole, asenapine, clozapine, lurasidone, olanzapine, paliperidone, quetiapine, risperidone, sertindole, and ziprasidone

**Table 2:** Hazard ratios (HR) and 95% confidence intervals (CI) for short-term acute kidney injury (90 days of follow-up). Cox regression, crude, and adjusted for covariates

	**Unadjusted**^**a**^ **HR (95% CI)**	**Partially adjusted**^**b**^** HR (95% CI)**	**Fully adjusted**^**c**^ **HR (95% CI)**
All SGAs	2.57 (1.80–3.68)	2.12 (1.43–3.15)	1.42 (0.89–2.27)
Olanzapine	5.57 (2.48–12.49)	4.44 (1.63–12.09)	3.50 (1.20–10.23)
Quetiapine	3.03 (1.77–5.20)	2.40 (1.34–4.29)	1.62 (0.81–3.26)
Risperidone	1.31 (0.65–2.62)	1.05 (0.50–2.21)	0.68 (0.28–1.64)

*SGA* second-generation antipsychotics

^a^Age and sex matched

^b^Adjusted for potential risk factors for acute kidney injury: NSAIDs, lithium use, alcohol abuse, drugs and diagnoses related to cardiovascular diseases, diabetes mellitus, hypertension, obesity, and cerebrovascular diseases, hepatic diseases and Parkinson’s disease

^c^Additionally adjusted for recent hospitalization

**Fig. 2:**
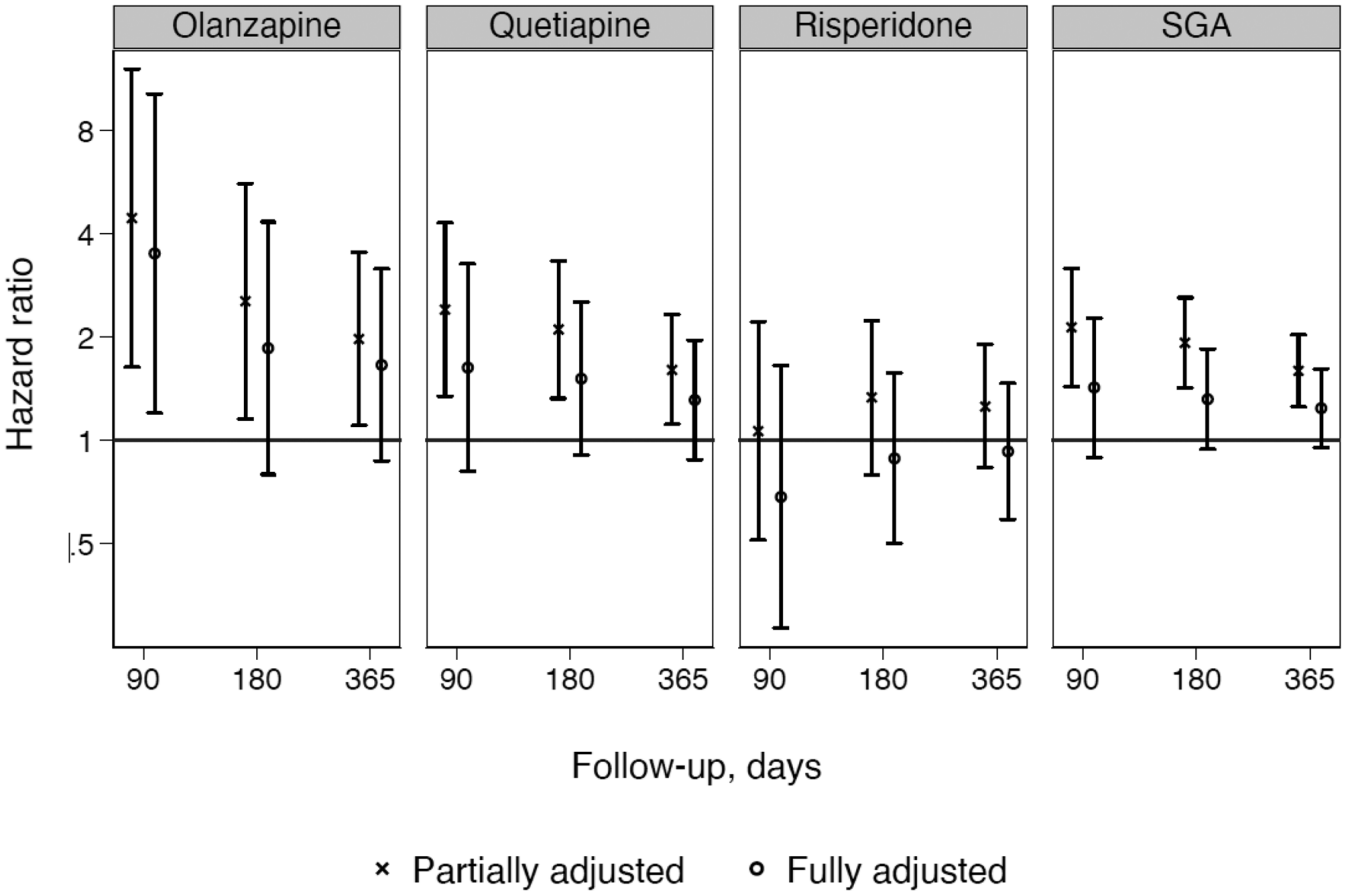
Plot of hazard ratio (HR) and 95% confidence intervals (CI) of the association between second-generation use and incident acute kidney injury

The 90-day IR of AKI was 4.38 per 1000 person-years among SGA users and 1.70 per 1000 person-years among the controls (for individual antipsychotics see Suppl. Table 3). This corresponds to an age-sex matched HR (95% CI) of 2.57 (1.80–3.68) (Table 2). In the partial adjustment model, the aHR was found to be 2.12 (1.43–3.15) for combined SGA users. In this model, both olanzapine with aHR 4.44 (1.63–12.09) and quetiapine with aHR: 2.40 (1.34–4.29) were significant, but not risperidone aHR 1.05 (0.50–2.21) (Fig. 2). However, in the fully adjusted model, including recent hospitalization, the association between combined SGA use and AKI was no longer significant with aHR 1.42 (0.89–2.27) (Fig. 2). In this model, the aHR was significantly elevated only for olanzapine with 3.50 (1.20–10.23) and not for quetiapine, 1.62 (0.81–3.26), or risperidone 0.68 (0.28–1.64).

In the sensitivity analyses, the fully adjusted analysis was repeated with longer follow-up time. The aHR decreased to 1.31 (0.94–1.84) for 6 months of follow-up and 1.24 (0.95–1.61) for 1 year of follow-up (Fig. 2). Additionally, we repeated the analysis with acute dialysis as outcome, where very few dialysis events were observed with no significant association (crude HR 1.23 (0.56–2.68) and aHR 0.67 (0.25–1.80)).

## Discussion

In this population-based cohort study of older patients, we found that olanzapine use was associated with a significantly increased AKI risk within 90 days of follow-up. The risk was also elevated for quetiapine, although not statistically significant, but not increased for risperidone. It is to be noted that the CI were wide and confounder adjustment had a large impact on the estimates, especially after adjusting for recent hospitalization.

A potential pathophysiological mechanism for the observed association could be differential affinity of SGAs to dopamine receptors D1–D5, in vascular smooth muscle and in the kidneys [36]. Dopamine has a renal vasodilatory action [37]. Most SGAs are antagonists of the dopamine D2-receptor, interacting with the sympathetic nervous system to regulate blood pressure [38]. Each of the SGAs’ selective dopamine receptor occupancy rate is different, and possibly dose-dependent [39]. Based on available information, olanzapine has the strongest affinity to the D4 and D1 receptors, thus interacting mostly with the renin–angiotensin–aldosterone system (RAAS) [38]. Hence, use of antipsychotic medication that block D2-receptors, e.g., risperidone and quetiapine, is associated with increased risk of developing hypertension and cardiovascular morbidity, whereas olanzapine use may have a more acute effect on blood pressure, electrolyte balance, and vascular resistance. Contrastingly, orthostatic hypotension is a known adverse drug reaction among older adults using both olanzapine, quetiapine, and risperidone [38, 40], possibly due to antagonism of D2-receptors.

In our study, the largest increase in AKI risk was observed after olanzapine exposure. Although a different affinity to dopamine receptor subtypes of olanzapine, quetiapine, and risperidone may be responsible, the exact pathophysiological explanation for this finding was not obvious from the literature. However, we hypothesize that olanzapine’s relatively selective antagonism of D4 and D1-receptors in the kidney could lead to an acute and local disruption in the fluid and electrolyte balance, which might contribute to altered homeostasis, blood pressure reduction, and reduced renal perfusion leading to AKI.

To our knowledge, there are only three previous studies investigating the association between SGA use and AKI [19–21], among which the studies of Hwang [19] and Ryan [20] are the most comparable ones to our study, with incident antipsychotic users and non-users (controls) as comparator and restricted only to older adults. Ryan et al. performed a replicative analysis of the Hwang study and additionally performed their own adapted analysis with additional confounding strategies [20]. Both studies excluded previous end-stage renal disease [19, 20]. In recent studies, pre-existing CKD was identified as a distinct risk factor for AKI, since both reduced glomerular filtration rate and elevated proteinuria were observed to be strongly associated with AKI [12, 41, 42]. Our study was restricted to SGA users and controls with no pre-existing renal events including CKD. This ensured that the majority of underlying renal-related risk factors of AKI were eliminated. In the Hwang study, individuals discharged from the hospital in the last 2 days prior to their index date were excluded to ensure new drug initiation in a non-hospitalized setting, but past hospital visits were adjusted for [19]. However, in our study, adjusting for recent hospitalization should mitigate the effect of other predisposing factors for AKI, e.g., surgery and sepsis [43]. The third study by Jiang et al. was very different in setting as compared to our study [21], due to their use of a prevalent-user design, ≥ 18 age group inclusion, and use of haloperidol as comparator. Despite these differences between studies, our study results of significantly elevated risk in olanzapine users and elevated risk in quetiapine users were compatible to those of Hwang, Ryan replicative, and Jiang. In contrast, we found no increased risk for risperidone. In Ryan’s replicative study, there was no association for any of the drugs.

### Strengths and limitations

Our study has important strengths of using Danish national registries that cover the entire population with limited or no selection bias [24, 25]. We used new-user study design with longer washout period than previous studies which eliminated potential bias from prevalent antipsychotic drug use. We used a short 90-day follow-up and excluded prior renal events (interventions, procedures, and complications to other diseases such as diabetes, hypertension, or cardiovascular disorders) to focus only on incident AKI [19]. The short follow-up should also reduce exposure misclassification bias related to drug discontinuation and switching. In addition to the age and sex matching, and adjustment for several factors associated with AKI, in the fully adjusted model, we also took into account recent hospitalization, which had a major impact on the results.

The main limitation of our study is its reliance on ICD-10 diagnosis for AKI. A recent study found that 9.7% of the older hospitalized adults developed AKI based on the serum creatinine criteria (9.4% developed AKI stage ≤ 2) [16]. Still, none of the cases was registered with AKI in the Danish National Patient Registry [16]. However, we did not use serum creatinine values due to the unavailability of nationwide laboratory data covering the entire study period. As a result, the current study’s AKI identification method using ICD-10 codes includes severe cases of AKI but not mild to moderate cases, hence may have poor sensitivity but the diagnosis is known to have high specificity [17, 35]. Regardless of the large study population, this low sensitivity of AKI diagnosis could have led to potential underestimation of AKI event rates and wider CIs. However, we believe that any misclassification of the outcome leading to hospitalization will be non-differential and would not lead to spuriously increased risk among antipsychotic users. To further explore the association, “acute dialysis” was considered as an outcome to represent dialysis-requiring AKI patients in the 90-day follow-up after SGA initiation. However, there were only few events with dialysis procedures carried out in this setting and no association was found between SGAs and dialysis.

As another limitation, we cannot rule out residual confounding in our analysis as unmeasured confounders may be present. We were unable to directly adjust for lifestyle factors such as alcohol use or obesity due to lack of such information in Danish registers. However, we adjusted for proxies of these lifestyle factors, which may have been registered only in patients with more severe conditions.

A third, potential limitation is that intention-to-treat analysis may lead to misclassification of exposure due to discontinuation of treatment related to non-adherence, which is common in patients treated with psychotropics [44], or due to adverse events [45]. However, in our study set-up by reducing the follow-up to 90 days, we also reduced the exposure misclassification bias. Another limitation of the study is that we could not investigate any dose-related effects, as the Danish National Prescription Registry does not contain information on indication, prescribed daily dose, or duration of drug use [24]. Finally, undiagnosed AKI in older adults could present symptomatically as confusion leading to treatment with an antipsychotic. Hence, protopathic bias cannot be ruled out.

### Implications and perspectives

Clinicians should be aware that AKI can occur after short-term olanzapine and quetiapine use in older adults. Thus, we recommend monitoring of serum creatinine in older patients treated with SGAs, in agreement with others [19, 22]. However, according to our study, serious AKI is a rare event and there is a need to replicate our findings in other settings, e.g., using other Nordic healthcare databases, if possible with data on serum creatinine measurements.

## Conclusion

Olanzapine use was associated with a significantly increased risk of AKI within 90 days of drug initiation. Among quetiapine users, the risk was elevated but not significant, and risperidone users had no significant association. Recent hospitalization was recognized as an important confounder for AKI. Despite the large study population, the AKI events were rare; hence, CIs were wide, and confounder adjustment had a major impact on results. The main limitations of the study are residual confounding and incomplete recording of AKI diagnoses.

## Supplementary information

Below is the link to the electronic supplementary material.

Supplementary file1 (DOCX 151 KB)
