# Supplementary material for: Incidence of hospital contacts with acute kidney injury after initiation of second-generation antipsychotics in older adults: a Danish population-based cohort study
Source: Eur J Clin Pharmacol. 2022 May 31;78(8):1341–9. doi: 10.1007/s00228-022-03339-6 (PMC9283184; doi:10.1007/s00228-022-03339-6)
Supplement: Supplementary file 1 — Supplementary file1 (DOCX 151 KB) [file 228_2022_3339_MOESM1_ESM.docx]

**Electronic Supplementary material for *European Journal of Clinical pharmacology***

Incidence of hospital contacts with acute kidney injury after initiation of second-generation antipsychotics in older adults: A Danish population-based cohort study

## Author list

Reeha Sharon^1^, Theis Lange^2^, Mia Aakjær^1^, Sarah Brøgger Kristiansen^1^, Morten Baltzer Houlind^3,4,5^, and Morten Andersen^1^

^1^Pharmacovigilance Research Center, Department of Drug Design and Pharmacology, University of Copenhagen, Copenhagen, Denmark.

^2^Section of Biostatistics, Department of Public Health, University of Copenhagen, Copenhagen, Denmark.

^3^Department of Clinical Research, Copenhagen University Hospital, Hvidovre, Denmark.

^4^The Capital Region Pharmacy, Herlev, Denmark.

^5^Department of Drug Design and Pharmacology, University of Copenhagen, Copenhagen, Denmark.

**Corresponding author**: Reeha Sharon, [reeha.sharon@sund.ku.dk](mailto:reeha.sharon@sund.ku.dk)

Pharmacovigilance Research Center, Department of Drug Design and Pharmacology, Faculty of Health and Medical Sciences, University of Copenhagen, Copenhagen, Denmark

# **Table 1**

**Definition of study drugs and co-medications by ATC codes**

|  | **ATC codes** |
| --- | --- |
| **Study drugs** | |
| **Most-used Second generation antipsychotics** | |
| Olanzapine | N05AH03 |
| Quetiapine | N05AH04 |
| Risperidone | N05AX08 |
| **Infrequently-used Second generation antipsychotics** | |
| Aripiprazole | N05AX12 |
| Clozapine | N05AH02 |
| Ziprasidone | N05AE04 |
| Paliperidone | N05AX13 |
| Amisulpride | N05AL05 |
| Asenapine | N05AH05 |
| Lurasidone | N05AE05 |
| Sertindole | N05AE03 |
| **Co-medications** | |
| Anti-depressants | N06A |
| Anti-epileptics | N03 |
| Anxiolytics | N05B |
| Anti-Parkinson drugs | N04 |
| **Antihypertensives** | |
| Antiadrenergic agents, centrally acting | C02A (excl. C02AC01, C02AC02) |
| Arteriolar smooth muscle, agents acting on | C02D |
| Low-ceiling diuretics, thiazides | C03A |
| Low-ceiling diuretics, excl thiazides | C03B |
| High-ceiling diuretics | C03C |
| Potassium-sparing agents | C03D |
| Diuretics and potassium-sparing agents in comb | C03E |
| Beta blocking agents | C07 |
| Calcium channel blockers | C08 |
| Angiotensin-converting enzyme inhibitors, plain | C09A |
| Angiotensin-converting enzyme inhibitors, combinations | C09B |
| Angiotensin ii receptor blockers, plain | C09C |
| Angiotensin ii receptor blockers, combinations | C09D |
| **Anti-obesity drugs** | |
| Antiobesity preparations, excluding diet products | A08 (excl. A08AA02, A08AA56) |
| **Cardiovascular drugs** | |
| Vitamin K antagonists | B01AA |
| Platelet inhibitors | B01AC |
| Direct thrombin inhibitors | B01AE |
| Direct factor Xa inhibitors | B01AF |
| Digoxin | C01AA05 |
| Statins | C10 |
| **Diabetes drugs** | |
| Antidiabetics | A10 |
| **Drugs known to induce renal events** | |
| Lithium | N05AN |
| Non-steroidal anti-inflammatory drugs | M01A (excl. M01AX05) |
| **Excluded drugs** | |
| First-generation antipsychotics | N05AA01, N05AA02, N05AA03, N05AA04, N05AB01, N05AB02, N05AB03, N05AB04, N05AC01, N05AC02, N05AC04, N05AD01, N05AD03, N05AD05, N05AD06, N05AD08, N05AF01, N05AF03, N05AF05, N05AG02, N05AG03, N05AH01, N05AL01 |

ATC: Anatomical Therapeutic Chemical classification [1]

# **Table 2**

**ICD-10 and NCSP codes for identifying diagnoses**

| **Diagnosis** | **ICD-10 codes/NCSP/SKS codes** |
| --- | --- |
| **Outcomes** | |
| Acute kidney injury | N00, N17 |
| Dialysis | SKS: BJFD0, BJFD00, BJFD01, BJFD02 |
| **Somatic disorders** | |
| Alcohol abuse | E244, F10, G31.2, G62.1, G72.1, I42.6, K29.2, K85.2, K86.0, R78.0, T51, Z50.2, Z71.4, Z72.1 |
| Cardiovascular diseases | G46, I09.9, I20-I25, I42.0, I42.5, I42.7-I42.9, I43, I46.0, I46.9, I47.0, I47.2, I48, I49.0, I49.1, I50, R93.1, T82.2, Z95.5, Z95.8-Z95.9 |
| Cerebrovascular diseases | G45, G45.4, H34.0, H34.1, I60-I69 |
| Congestive heart failure | I25.5, I50 |
| Diabetes | E10-E14, G59.0, G63.2, H28.0, H33.4, H36.0 |
| Hepatic diseases | B16-B19, I85, R17, R18, R16.0, R16.2, B94.2, E83.0, E83.1, K70-K77 |
| Hypertension | I10-I15 |
| Obesity | E65, E66, E78, R63.5 |
| Vascular diseases | I70.0-I70.2, I70.8, I70.9, I71, I73.1, I73.8, I73.9, I77.1, I79, K55.1, K55.8, K55.9 |
| **Psychiatric and neurological disorders** | |
| Alzheimer’s disease | G30 |
| Anxiety | F40-F42, F43.0, F43.1 |
| Schizophrenia and schizoaffective disorders | F20-F25, F28, F29 |
| Bipolar disorder | F30, F31 |
| Dementia | F00-F03, F05.1, F09, G31, R54 |
| Depression | F32, F33, F34.1 |
| Epilepsy | G40, G41 |
| Mood disorders | F34, F38, F39 |
| Other psychotic disorders (not categorised) | F04-F07 |
| Parkinson's disease | F02.3, G20-G26 |
| **Excluded diagnosis** | |
| Cancer | C00-C97 |
| Chronic kidney disease | I12.0, I13.1, I13.2, N01, N04, N05, N06, N08, N14.1, N14.2, N16.8, N18, N19, N25.0, N25.1 |
| Renal complications | ICD10: E10.2, E11.2, E13.2, E14.2, I12.9, I13.0, I13.9, I15.1, I70.1, N26, N27, Z49.0, Z49.0, Z49.2, Z94.0, Z99.2  NCSP: KAS |

ICD-10: International Classification of Diseases [2]; NCSP: Nordic classification of surgical procedures [3]; SKS: Danish Health Care Classification System (Sundhedsvæsenets Klassifikations System) [4]

# **Fig.1 Graphical depiction of time windows for inclusion, exclusion, covariates and follow-up**


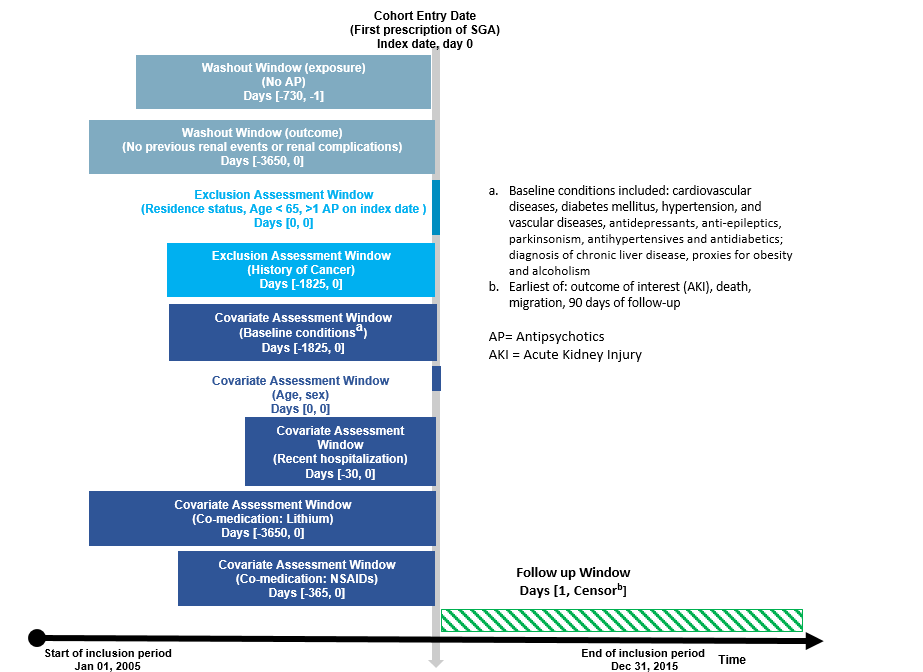


Figure based on Schneeweiss et al [31]. Licensed under CC BY, can be found at <https://presc.sdu.dk/repeat-diagrams/.up>

# **Fig. 2 Nordic Common Data Model (NCDM) and analytics framework**

The Nordic Common Data Model (NCDM) is a structured database adapted to Nordic healthcare registers and used for pharmacoepidemiological research [5, 6]. An earlier version was developed at the Karolinska Institutet for the CARING project [7]. The Pharmacovigilance Research Center (PVRC) at the University of Copenhagen (UCPH) maintains a newer version and a framework of modular analysis programs allowing for rapid analytics. We used the below structure from the NCDM/UCPH framework to create the analysis datasets for this study. The framework converts raw data (population, hospital encounters, diagnosis, procedures, and drugs used) from the registries to the NCDM database and generates final analysis datasets and result tables, along with automated quality checks for each of the steps involved. Researchers interested in the NCDM/UCPH framework programs are welcome to reach out to head of the PVRC: Prof. Morten Andersen at [morten.andersen@sund.ku.dk](mailto:morten.andersen@sund.ku.dk)


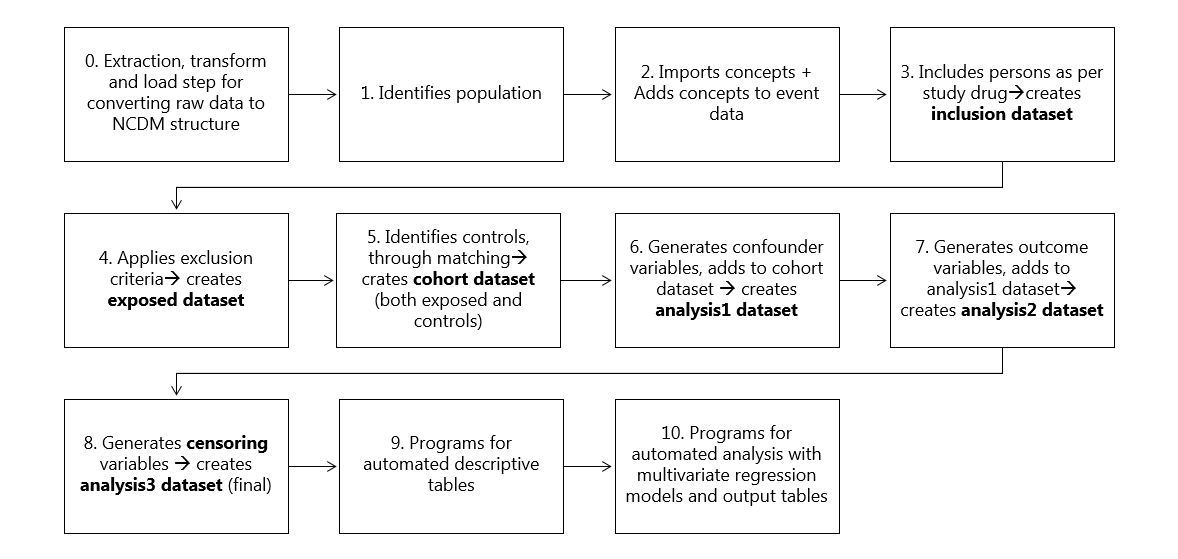


# **Table 3**

Number of events (N), person-years and incidence rates (IR) per 1000 person-years for acute kidney injury. Most-used second generation antipsychotics (SGAs)

|  | **SGA users** | | | **Controls** | | |
| --- | --- | --- | --- | --- | --- | --- |
|  | **N** | **Person-years** | **IR** | **N** | **Person-years** | **IR** |
| Olanzapine | 9 | 1248 | 7.21 | 17 | 13,227 | 1.29 |
| Quetiapine | 17 | 3606 | 4.71 | 58 | 37,451 | 1.55 |
| Risperidone | 9 | 3344 | 2.69 | 73 | 35,835 | 2.04 |

**References to supplementary material**

1 WHO (2021) ATC Index with DDDs. <https://www.whocc.no/atc_ddd_index/> Accessed 10 February 2021.

2 WHO (2019) International Classification of Diseases (ICD)10th Revision. <https://icd.who.int/browse10/2019/en#/J09> Accessed 12 February 2021.

3 NOMESCO (2010) NOMESCO Classification of SurgicaL Procedures (NCSP),version 1.15. <http://norden.diva-portal.org/smash/get/diva2:970547/FULLTEXT01.pdf> Accessed 13 July 2021.

4 Danish-Health-Authority Danish Health Care Classification System (Sundhedsvæsenets Klassifikations System (SKS)). <https://medinfo.dk/sks/brows.php> Accessed 29 September 2021.

5 Andersen M, Thinz Z, Citarella A, Bazelier M, Hjellvik V, Haukka J, de Groot M, Furu K, Vestergaard P, De Bruin M (2015) Implementing a Nordic Common Data Model for register-based pharmacoepidemiological research (D19). Norsk Epidemiologi 25 (0) DOI 10.5324/nje.v25i0.1933

6 Cohen JM, Cesta CE, Kjerpeseth L, Leinonen MK, Hálfdánarson Ó, Karlstad Ø, Karlsson P, Andersen M, Furu K, Hjellvik V (2021) A common data model for harmonization in the Nordic Pregnancy Drug Safety Studies (NorPreSS). Norsk Epidemiologi 29 (1-2) DOI 10.5324/nje.v29i1-2.4053

7 But A, De Bruin ML, Bazelier MT, Hjellvik V, Andersen M, Auvinen A, Starup-Linde J, Schmidt MK, Furu K, de Vries F, Karlstad Ø, Ekström N, Haukka J (2017) Cancer risk among insulin users: comparing analogues with human insulin in the CARING five-country cohort study. Diabetologia 60 (9): 1691-1703 DOI 10.1007/s00125-017-4312-5
